# Tracing the Primordial Chemical Life of Glycine: A Review from Quantum Chemical Simulations

**DOI:** 10.3390/ijms23084252

**Published:** 2022-04-12

**Authors:** Albert Rimola, Nadia Balucani, Cecilia Ceccarelli, Piero Ugliengo

**Affiliations:** 1 Departament de Química, Universitat Autònoma de Barcelona, 08193 Catalonia, Spain; 2 Dipartimento di Chimica, Biologia e Biotecnologie, Università di Perugia, Via Elce di Sotto 8, 06123 Perugia, Italy; nadia.balucani@unipg.it; 3 Osservatorio Astrosico di Arcetri, Largo E. Fermi 5, 50125 Firenze, Italy; 4 CNRS, Institut de Planétologie et d’Astrophysique de Grenoble (IPAG), Université Grenoble Alpes, 38000 Grenoble, France; cecilia.ceccarelli@univ-grenoble-alpes.fr; 5 Dipartimento di Chimica and Nanostructured Interfaces and Surfaces (NIS) Centre, Università degli Studi di Torino, Via P. Giuria 7, 10125 Torino, Italy; piero.ugliengo@unito.it

**Keywords:** astrochemistry, prebiotic chemistry, interstellar grains, primitive Earth, computational chemistry, surface modelling, potential energy surfaces, metadynamics

## Abstract

Glycine (Gly), NH_2_CH_2_COOH, is the simplest amino acid. Although it has not been directly detected in the interstellar gas-phase medium, it has been identified in comets and meteorites, and its synthesis in these environments has been simulated in terrestrial laboratory experiments. Likewise, condensation of Gly to form peptides in scenarios resembling those present in a primordial Earth has been demonstrated experimentally. Thus, Gly is a paradigmatic system for biomolecular building blocks to investigate how they can be synthesized in astrophysical environments, transported and delivered by fragments of asteroids (meteorites, once they land on Earth) and comets (interplanetary dust particles that land on Earth) to the primitive Earth, and there react to form biopolymers as a step towards the emergence of life. Quantum chemical investigations addressing these Gly-related events have been performed, providing fundamental atomic-scale information and quantitative energetic data. However, they are spread in the literature and difficult to harmonize in a consistent way due to different computational chemistry methodologies and model systems. This review aims to collect the work done so far to characterize, at a quantum mechanical level, the chemical life of Gly, i.e., from its synthesis in the interstellar medium up to its polymerization on Earth.

## 1. Introduction

There is clear evidence that the Universe contains a rich chemical diversity and complexity. Since the detection of the first interstellar polyatomic molecule, i.e., NH_3,_ in 1968 [1], astrochemistry has experienced vertiginous growth, and it is, at present, a mature field of astrophysics.

Spectroscopic and photometric observations show that the space between stars is not empty but filled with diffuse matter, the interstellar medium (ISM). The ISM consists of gas and submicron-sized grain particles mixed together, which are not evenly distributed in the galaxy but are aggregated in clouds of gas, dust and ice [2,3,4]. Radio to near-infrared observations of the gaseous component have allowed the detection of more than 250 gas-phase molecules in the millimeter and submillimeter spectral regions [5]. Mid-infrared absorption has been used to probe the existence, composition and structural state of the interstellar grains. In diffuse clouds (with temperature 50–100 K and gas density 10–10^2^ cm^–3^), grains consist of amorphous silicates or carbonaceous materials [6,7], while in dense cold clouds (with temperature 5–10 K and gas density 10^4^–10^5^ cm^–3^) these refractory materials are covered by ice mantles, mainly made of H_2_O, and less abundant species, such as CO, CO_2_, NH_3_ and CH_3_OH [8].

The chemistry involved during the formation of a solar-type planetary system from a primordial interstellar cloud is of great significance. Indeed, astronomical observations show that the physical evolution of a nascent solar-type system goes hand-in-hand with its chemical evolution [9], in which, at each step, more complex molecules form. In turn, the chemical evolution during the first phases of a solar-type planetary system formation could ultimately be connected with the origin of life [10,11]. The increase in molecular complexity can be summarized in three major steps: (i) formation of simple molecules, such as H_2_ (the simplest ever molecule), H_2_O, NH_3_ or CH_3_OH, during the prestellar phase; (ii) synthesis of interstellar complex organic molecules (iCOMs [12]), which are made up of 6–13 atoms where at least one is C, such as CH_3_CHO or NH_2_CHO, during the protostellar phase; and (iii) enhancement of the chemical complexity, with the production of some molecules with biological relevance, such as amino acids, nucleobases and sugars, during the final planet formation phase [9]. Remarkably, the chemical richness observed in solar-type planetary systems is not limited to local galactic star formation. Indeed, clouds in external galaxies are also made up of gas enriched with molecules (a remarkable fraction, ca. 33%, of the known interstellar molecules have also been detected towards external galaxies [5]) and grains (also consisting of silicates, carbonaceous grains and ice mantles [13,14]). Therefore, the same reactions responsible for the chemical richness of our galaxy also take place in external galaxies, demonstrating that chemistry, which is ultimately at the base of terrestrial life, is universal, as indicated by the Nobel prize C. De Duve [15].

Simple molecules and iCOMs have been detected in different astrophysical environments in the early stages of star formation (i.e., prestellar cores, protostellar envelopes and protoplanetary disks). Reaction chains taking place in the gas phase can explain the formation of some of these compounds but not all of them. Indeed, grains, besides their role in absorbing the interstellar UV photons and protecting molecules from photolysis, can also act as helpers in the formation of key species that cannot be efficiently synthesized by gas-phase reactions. Examples of species that are synthesized by surface reactions (evidenced by experimental and theoretical investigations) are H_2_ [16,17,18,19,20], H_2_O [21,22,23,24,25,26], CO_2_ [27,28] and CH_3_OH [27,28,29,30] as simple molecules, and some iCOMs such as CH_3_CH_2_OH [31] and, perhaps, NH_2_CHO and CH_3_OOCH [32,33,34,35,36,37,38,39]. Molecules of more enhanced complexity than iCOMs are hitherto exclusively identified in some comets and meteorites [40,41].

Two reasons could explain this exclusivity. On the one hand, the detection of large molecules in the ISM, which is only possible via the identification of radio to microwave rovibrational spectral lines, is hampered by the lower abundance and the larger number of transitions in species of increasing complexity. The result is that increasingly complex molecules have weaker and weaker lines, and, ultimately, their lines form a grass in the observed spectra, where it is impossible to uniquely identify a complex molecule. On the other hand, meteorites are fragments of asteroids that, when they fall on Earth, can be studied in terrestrial laboratories. Similarly, in situ (space mission) observations of comets allows us to identify rather complex molecules. In addition, alteration processes occurring in asteroids probably form complex species [42]. Indeed, hydrothermal processes can give rise to a net increase in molecular complexity because the asteroidal material, called “parent material”, undergoes a variety of organic reactions in solution, yielding the formation of a new generation of more complex organic molecules [43,44,45]. Moreover, the inorganic solid components of these bodies (i.e., minerals, rocks and ices) have also been advocated to act as catalysts in the formation of these compounds [46,47,48].

Some comets are remnant planetesimals that did not end up on planets and were scattered in and from the cold outer regions of the solar system. Space- and Earth-based investigations show that comets contain organic entities [49], which can be refractory in nature (and hence the suspicion that they are macromolecular organic polymers [50]), or volatile compounds [51,52,53], like small aliphatic hydrocarbons (e.g., CH_4_, C_2_H_6_ and C_2_H_2_) and simple aldehydes (e.g., H_2_CO, CH_3_CHO, NH_2_CHO), alcohols (CH_3_OH), carboxylic acids (HCOOH) and nitriles (CH_3_CN). Among the meteorites, the carbonaceous chondrites (CCs) are the most pristine, less altered ones and are considered similar to the materials forming the original planetesimals. CCs contain large fractions of organic components [41,54], which can be insoluble organic material (IOM, a mixture of kerogen-like material and polycyclic aromatic hydrocarbons) and soluble organic compounds, which can be as wide and diverse as amines, amides, alcohols, aldehydes, ketones, carboxylic and hydroxycarboxylic acids, sulphonic and phosphonic acids, aliphatic and aromatic hydrocarbons, and heterocyclic compounds. These organic compounds are important in terrestrial biochemistry as they could have been exogenously delivered to the early Earth, hence contributing to the emergence of the first prebiotic building blocks of life.

Among the different extraterrestrial organic compounds with biological relevance, the amino acid glycine (Gly) enjoys a central position. All the α-amino acids have a general formula of NH_2_CHRCOOH, in which NH_2_CHCOOH is the backbone chain (common for all the α-amino acids) and R is the lateral chain (allowing one amino acid to be differentiated from the others). Gly is the simplest amino acid because R=H, and its presence has been confirmed both in the Stardust’s Wild 2 [52,53] and Rosetta’s 67P [51] comets as well as in several CC meteorites [41,54,55]. Because of its chemical “simplicity” and its biological significance, Gly is a paradigmatic case to trace the sequential path of “formation → transportation → exogenous delivery to a primordial Earth” of essential biomolecular building blocks that could have played a crucial role in the emergence and evolution of biochemical systems in a prebiotic era. For the particular case of Gly (as well as for the other amino acids), condensation reactions between them lead to the formation of peptides and, ultimately, proteins.

The first step, the Gly formation, could take place in the early phases of the solar-type planetary system formation. In principle, Gly can be synthesised in the gas phase and/or on the surfaces of the dust grains [56]. Despite various propositions of gas-phase routes of Gly formation, none have so far been found to be efficient. The studied neutral-neutral and ion-neutral reactions have large activation barriers [57,58]. Thus, the alternative is its formation on the grain surfaces. In terrestrial laboratories, Gly has been successfully synthesized in experiments based on the energetic processing of ices [59,60,61,62,63,64], whose composition is presumably similar to the interstellar ices. These experiments often use UV or proton/electron irradiation to simulate the stellar UV field and the cosmic rays that permeate the galaxy, although in quantities several orders of magnitude larger in order to have detections. As an alternative to these energetic processes, the non-energetic addition of atoms and radicals to ice mantle analogues also results in the formation of Gly [65,66]. However, one should bear in mind that, despite these positive results, interstellar Gly has not been detected yet, in spite of the numerous attempts [67,68,69,70]. A possible reason for the non-detection, in addition to the intrinsic difficulty to detect weak lines from such large and low-abundance species (see above), is that, if formed on the grain surfaces, the mechanism which would then liberate Gly into the gas phase could destroy it, as Gly is a fragile molecule [71].

Astrochemistry investigations have been traditionally based on a multidisciplinary approach, in which astronomical observations are combined with astrochemical modelling and laboratory experiments. However, this approach holds several limitations, especially when grain surface chemistry is concerned. Indeed, spectroscopic astronomical observations detect molecules and their quantity, but they do not tell us how they are formed, whether in the gas phase or on the grain surfaces, and, in the latter case, whether the presence of grains would be mandatory. Astrochemical models are used to reproduce and rationalize the observations; however, the uncertainties associated with the various parameters (reaction rate coefficients, species diffusion on the surfaces, etc.) used as input data cause the predictions to also be uncertain [72]. Laboratory experiments have been used to characterize the nature of the formed products, but the details of the reaction mechanisms and the exact role played by the grains cannot be elucidated [73], causing controversies in the proposed mechanisms. Such limitations can partly be alleviated by using a fourth pillar of investigation: quantum chemical simulations (QCs), as they can provide unique, quantitative information on processes of astrochemical (and prebiotic as well) relevance [35,74,75], such as molecular mechanistic steps and the associated structure and energetics at an atomic scale.

In this comprehensive review, the likely interstellar origin of Gly and its subsequent evolution to form peptides in a primitive Earth are reviewed from a computational chemistry viewpoint. The aim is to provide a plausible chemical path of Gly, i.e., from its formation, passing through its transportation and delivery to the early Earth, up to its biochemical evolution under prebiotic conditions, validated by works that used QCs to investigate events involved in this sequence. Special consideration is paid to those processes in which the role of solid-state surfaces plays an essential role. Gly is here considered as a paradigmatic test case, with the good understanding that other important biomolecular building blocks (e.g., other amino acids, nitrogenous bases and sugars) can follow similar paths.

The review is organized as follows. In Section 2, the computational framework in terms of quantum chemistry methods and surface modelling is briefly exposed. Section 3 is devoted to the formation of Gly in different astrophysical environments. Section 4 focuses on the transportation of Gly and its delivery to the early Earth by cometary and meteoritic materials. Section 5 addresses the chemical evolution of the delivered Gly in a prebiotic context, particularly the condensation reaction between two Gly molecules forming a peptide. Finally, Section 6 presents the main conclusions alongside some future perspectives that can help make further advances in this branch of the primordial chemical evolution by means of computational chemistry simulations.

## 2. Computational Framework

### 2.1. Quantum Chemical Methods and Basis Sets

Accurate calculations for reactivity (i.e., dealing with bond formation and/or breaking) require the use of quantum chemistry methods. Most of the time we are dealing with conditions and molecular entities in which the solution of the time-independent, non-relativistic electronic Schrödinger equation within the Born–Oppenheimer approximation is good enough (these calculations are often called electronic structure calculations). Quantum chemical methods can be classified into two major groups, based respectively on: (i) the wave function (WF) and (ii) the electron density through the density functional theory (DFT). WF methods are based on the resolution of the electronic Schrödinger equation for an N-electrons wave function. DFT methods, instead, focus on the Kohn–Sham rigorous theorem [76], establishing a one-by-one relationship between the energy of the non-degenerate electronic ground state and its corresponding electron density ρ(r) through an unknown (but formally proved to exist) functional F[ρ(r)] of the density itself.

The simplest WF-based method is the Hartree–Fock (HF) method, in which the WF is represented by the best set of mono-electronic spin-orbitals within a single Slater determinant representation of an N-electron system. This relatively simple description of the WF allows the HF equations to be solved in a highly efficient way by modern computer programs, at the price of the limited accuracy of the physicochemical predictions. The reasons for the limited accuracy lay in the instantaneous electron-electron correlation, lost within the HF mean-field approximation. This leads, among many other drawbacks, to a bad description of radical species, H-bond and charge transfer processes; besides, London dispersion interactions are also entirely missing within the HF approach.

Despite these inaccuracies, the HF wave function is used as the zero-order term in the expansion of the multi-electron WF in terms of a very large (in principle infinite) number of Slater determinants envisaging all possible electron configurations. This approach is known as the configuration interaction (CI) method. Other techniques, based on the coupled-cluster (CC) ansatz, build up the multi-electron WF using the exponential cluster operator to account for electron correlation. CC is much more effective than CI in including a larger portion of the electron correlation, particularly within its CCSD(T) incarnation, nowadays known as the “golden standard” [77,78,79] in quantum chemistry calculations for its accuracy and generality in treating a large variety of molecular properties. Unfortunately, post-HF methods are prohibitively expensive and thus unpractical when dealing with the reactivity of large molecular systems or modeling molecule/surfaces interactions, as the computational resources grow exponentially with the system size. Nevertheless, an outstanding effort has been provided by the computational chemistry community to make very efficient computer programs, like, for instance, ORCA and Cfour, which can efficiently compute CCSD(T) by exploiting either new approaches based on local orbitals (like in the DLNPO-CCSD(T) method) or by exploiting the massive parallelism of the high-performance computing architectures. In practice, post-HF methods are mostly used in benchmarking more approximate methods (e.g., DFT, see below) to assess their suitability to simulate specific physicochemical observables of the target systems.

As already pointed out, an alternative to the many-electron wave function is to consider the electron density, through which the total energy of the ground state can be evaluated by a universal and exact mathematical functional, at least in principle. Kohn and Sham demonstrated that the energy functional does indeed exist [80], but its explicit mathematical form is, unfortunately, unknown. Attempts to discover the exact energy functional are at the roots of the density-functional theory (DFT). A huge effort from the theoretical chemistry community has been put forward to improve the functional forms by comparing the results either with accurate experiments or with the CCSD(T) ones, when feasible. While a large number of good functionals are nowadays available, it becomes a tour de force for a computational chemist to pick up the one giving reasonable accuracy for a broad class of observables, avoiding the risk of cherry-picking a functional apt for only a specific set of predictions (see below). DFT methods are attractive with respect to WF-based ones because they include a considerable fraction of electron correlation at a slightly lower cost than the standard HF. Because of its applicability to both large molecular systems as well as extended (i.e., periodic) systems, DFT has become the workhorse of quantum chemical simulation. The most common functionals encoded in periodic simulation programs (mostly using plane-wave basis set, see below) are those based on the generalized gradient approximation (GGA) of the electron density, such as PBE [81], PW91 [82] or BLYP [83,84]. Hybrid DFT functionals (e.g., B3LYP [85], PBE0 [86] or BHLYP [87]) contain in their definition a percentage of exact exchange from the HF-WF solution, and their implementation is more efficient using localized basis functions (usually Gaussian-type orbitals) than plane waves (see below). Meta-GGA (e.g., TPSS [88] or M06L [89]) and hybrid-meta DFT methods (e.g., M06 or M062X [90]) include a term explicitly depending upon the kinetic energy density and/or its Laplacian, with the aim to improve the performances of the GGA and hybrid functionals. Hitherto, the “best functional for everything” has not been developed yet [91], and the current strategy is to identify the functional that better describes the features of the studied system (e.g., thermochemistry, kinetics, noncovalent interactions, etc.) by means of a benchmarking study (with some warning, see above).

The oldest and, therefore, more widespread functionals usually do not account for dispersion-based (London) interactions [92,93,94]. This is a serious limitation because they are essential to properly describe intermolecular interactions present, for instance, between two reactive species or in adsorbate/adsorbent complexes. A pragmatic solution is to supplement the DFT electronic energy with the dispersion contribution through an a posteriori correction term (D) based on the classical atom-atom additive empirical potential based on the old London formula [95]. This DFT-D scheme was proposed by Grimme and co-workers and sequentially improved to account for system specificity (D [96], D2 [97], D3 [98,99] and D4 [100]). The DFT-D approach keeps the original accuracy of the DFT functional for short-range interactions (bond breaking/making), significantly improving, at the same time, the London-dominated interactions at an almost zero computational cost. Proper damping functions have been adopted to remove the London term at a short range.

For system sizes too large to limit the DFT applicability, the alternative strategy is to use semiempirical methods (SMs). Historically, SMs are a simplified version of the HF method, in which the expensive bi-electronic integrals are ignored and replaced by empirical parameters derived either from experiments or from a higher level of theory on a set of target molecules (training set). Because of these simplifications, SMs are much faster than HF, allowing the treatment of hundreds/thousand atoms. Nonetheless, the validity and accuracy of the SMs outside the training set may be critical. Historically, SMs derived from HF were AM1 [101] and MNDO [102], while the PMx family [103,104,105]) has constantly been improved up to the PM7 model. Simplification of the DFT theory is also possible through the tight-binding approach such as SCC-DFTB [106,107]. More recently, the SM-DFTB GFN-xTB family of methods [108,109,110] has been proposed by the Grimme group and is gaining momentum due to its broad applicability to a large number of organic and metalorganic systems reaching surprisingly high accuracy at a computational cost inferior to the best PM7 method.

In parallel to these developments, efforts to reach high accuracy comparable to post-HF at a reasonable cost have also been performed, giving rise to what are called “composite methods”. The main feature of these methods is that they combine the results obtained with different quantum chemical theory levels. For instance, structures can be computed at a reasonable cost/effective level (e.g., DFT), while the energies are computed through single-point calculations at a higher level of theory (e.g., post-HF). Additionally, zero-point energy corrections can be estimated using a lower level of theory. Among the most popular composite methods, one can find the Gn and CBS families and their derivatives, such as G3MP2 [111] or CBS-QB3 [112,113].

The described methods are all based on some function representation of the spin orbitals contributing to the description of the wave function or the electron density. Usually, two kinds of mathematical functions are used to expand the orbitals in terms of a set of basis functions: (i) localized atom-centered functions of Gaussian-type orbitals (GTOs) for WF and DFT or Slater-type orbitals (STO) for SMs, and (ii) plane waves (PWs), which are fully delocalized. GTOs are common in molecular calculations, while PWs are the natural choice for describing crystals extending in the three periodic directions, as PWs are intrinsically periodic. Therefore, when PWs are used even for molecules, one should enforce a fake periodicity to the system, as PWs fill in the whole space available. They are ideal for treating metal bulks and surfaces due to the electron delocalization of these systems. For molecules or crystalline materials of microporous nature (like zeolites, MOF, nanotubes, etc.), PWs become impractical as they describe mainly the empty space in the unit cell. GTOs, on the contrary, are ideal for treating well-localized systems, like molecules, oxides and covalent crystals, provided that a large enough number of GTOs is used to provide enough flexibility to describe electron deformation. Unfortunately, GTO calculations suffer from the basis set superposition error (BSSE). BSSE affects all calculations, including the potential energy surface (PES) of chemical reactions and especially the evaluation of structures and interaction energies of intermolecular complexes as well as adsorbate/surfaces systems. Briefly, the BSSE is due to the incompleteness of the basis set, causing an unbalanced description of the wavefunction (and, therefore of the energy) of molecular complexes or surface/adsorbates with respect to their free constituents. Fortunately, a computational recipe known as “the counterpoise method” [114] is available to reduce its effect on both structures and energies. In contrast, properties computed with the PW basis set do not suffer from BSSE, as PW-based calculations are always carried out within periodic boundary conditions, even for isolated molecules: they uniformly fill the unit cell where the systems are defined and, therefore, the basis set density is the same for a molecular complex or its separate constituents. Finally, as already mentioned above, computer codes based on PWs are more efficient than those adopting GTOs for energy and force calculations when GGA functionals are employed, while the reverse is true for hybrid functionals (PBE0, B3LYP, etc.). This is because the calculation of the HF-exact exchange using PWs can be more than one order of magnitude slower than when adopting GGA functionals.

### 2.2. Potential Energy Surfaces (PESs) and Thermochemical Corrections

The energy features of a chemical reaction (i.e., reaction energies, energy barriers, and, when occurring on surfaces, adsorption/desorption energies) can be characterized by its PES. Thus, PESs describe the evolution along a specific reaction coordinate of the energy of a reaction as a function of the geometries of the reacting species at each point of the reaction coordinate (energy profile). The most relevant points of the PESs (computed by means of any of the quantum chemical methods explained above) are the stationary points, i.e., points in which the gradient of the energy is zero. Stationary points have a physical meaning: minima are stable species and correspond to reactants, products, and intermediates, while first-order saddle points correspond to transition state (TS) structures. A TS is the highest energy structure connecting two minima (e.g., between reactants and products), thus representing the minimum energy path to go from one minimum to the other.

The nature of the stationary points is characterized by the eigenvalues of the Hessian matrix, the second derivatives of the potential energy with respect to the atomic positions. Hessians with all positive eigenvalues characterize minima of the PES while one imaginary eigenvalue defines a TS, the associated eigenvector providing the direct line connecting the two minima. As the eigenvalues are related to the normal modes of vibration, in practice, by computing the vibrational frequencies of the stationary points, we can characterize their nature.

Vibrational frequencies, using the harmonic approximation, are key to computing the vibrational partition functions along the translation (through the system mass), rotational (through the system structure), and electronic (from the system electronic states) to get the thermochemical corrections to the potential energy (i.e., corrections for zero-point energy, internal energy, enthalpy, entropy, and Gibbs free energy) through the application of the classical thermodynamics statistics formulae [115]. The absolute free energy of a species at a given temperature T, G(T), is, therefore:

G(T) = E + ZPE + U(T) + PV(T) − TS(T),(1)

in which E is the potential energy of the species, ZPE the zero-point energy, U(T) is the thermal contribution to the internal energy, and P, V and S(T) are the volume, pressure and entropy, respectively. This latter is obtained by considering the vibrational states (usually through a harmonic model), the rotational states (usually through the rigid rotor model) and the translational states. Under the interstellar conditions, the P, V and S(T) contributions are negligible due to the extremely low temperature and pressures. By introducing the corrections into the PESs, one can get approximated ZPE-corrected energy profiles as well as free energy profiles of the reactions, the values of which can be used to derive elementary rate constants by applying kinetics formalisms.

### 2.3. Molecular Dynamics and Metadynamics

PESs are built from the electronic structure calculation of the different stationary points, which are structures in which atoms are considered stationary (besides the ZPE motion, vide supra). Thus, in these calculations, dynamic effects inferred by the temperature are not considered, and hence they are called static calculations.

Molecular dynamics simulations (MDs) must be employed to account for dynamical effects. They are also based on the Born–Oppenheimer approximation (separation of the electrons and nuclei degrees of freedom), in which the nuclei equations of motion are propagated classically following Newton’s equation, in which the nuclei velocities are a function of the macroscopic temperature of the system. The forces acting on the nuclei are the opposite of the first derivative of the potential energy E, computed at the DFT level at each time step of the system evolution. To ensure proper energy conservation (in the case of the microcanonical system simulation), the integration time step should be less than 1 fs. These kinds of simulations are referred to as ab initio molecular dynamics simulations (AIMDs) [116]. As AIMDs are based on a DFT energy PES, they envisage the formation and breaking of chemical bonds. However, simulations of chemical reactions with standard AIMDs are hampered by the limitation of practical simulation timescales. That is, a chemical reaction is a rare event (due to the need of surmounting one or more energy barriers) that take place in much longer timescales than those provided by AIMDs (on the order of picoseconds). This drawback can be alleviated by adopting metadynamics [117,118]. It is a powerful computational technique that allows the sampling of free energy surfaces on selected degrees of freedom that govern the chemical reaction, which are called collective variables (CVs). The sampling is facilitated by the introduction of an external bias potential acting on the CVs. This external potential is a sum of Gaussian functions deposited along the system trajectory in the space of the CVs. The resulting deformed PES has reduced (up to zero) kinetic barriers, which can be easily overcome during the simulation time. Hence, metadynamics are informally described as “filling the free energy wells with computational sand”.

The selection of appropriate CVs is crucial, as they determine the proper evolution of the reaction along the trajectories. They are the analogues of the reaction coordinates in energy profiles. However, at variance with static calculations, CVs are described as mathematical functions representing the change that drives the reaction. Examples of CVs are interatomic distances, coordination numbers, or normal modes.

### 2.4. Surface Modeling

Modelling surfaces requires the adoption of a model representative of the real surface. To this end, two strategies can be adopted: the periodic approach and the cluster approach [74,75,119,120]. In the periodic approach, a slab of finite thickness is cut out from the bulk (along specific Miller indexes) of the system under study. The resulting unit cell containing the most relevant surface features (normally adsorptive/catalytic sites) is repeated to an infinite 2D slab model. Convergence of the results (surface energy, structures, surface electrostatic potential, etc.) should be checked against the slab thickness. In contrast, the cluster approach consists of using a finite block of atoms cut out from the 2D slab model properly saturated at the frontiers of the block, arriving at a molecular system.

Let us start with the periodic approach. A perfect 3D bulk crystalline solid (i.e., without defects, impurities and any other irregularity) is defined by a crystallographic unit cell envisaging six independent parameters, i.e., **a**, **b**, **c** cell vectors and the intra-vectors α, β and γ angles, whose atomic content is repeated along a lattice vector T = m·**a** + n·**b** + p·**c** where m, n and p are integer numbers spanning the [−∞, +∞] range. Thus, the lattice vector T enforces the translation symmetry of the crystal. Therefore, the electron density ρ(r) of the crystal unit cell is replicated under the action of the lattice vector **T** with the translated ρ(**r** + **T**) satisfying the relation: ρ(**r** + **T**) = ρ(**r**), also referred to as the periodic boundary condition (PBC). It is worth noting that the PBC can also serve to simulate amorphous systems. However, since in real amorphous materials, the long-range order is missing, it is customary to define a large crystal unit cell containing enough atoms to be representative at least of the local disorder of the amorphous material. The application of the PBC will generate replicas of the unit cell, ultimately defining a crystal of amorphous unit cells with exactly the same amount and kind of disorder. Clearly, at variance with true crystalline systems, in which symmetry elements relate atomic positions in the unit cell, for amorphous systems, only the identity operation is left, with a consequent increase in the computational resources.

As anticipated before, a 2D slab model can be adopted for further studies, and the convergence of the surface energy, E_S_, should be ensured. This quantity represents the energy penalty suffered by the slab when it is detached from the crystal bulk, and it is defined as:

E_S_ = [E(slab) − N·E(bulk)]/(2·A), (2)

in which E(slab) is the energy of the unit cell of the slab model, E(bulk) is the corresponding one for the bulk, N is the number of unit cells in the slab, and A is the surface area. The factor 2 accounts for the two surfaces defining the slab model, assumed to be of identical nature. This last point is very important because the slab model should not exhibit an electric dipole moment across the slab, which will introduce catastrophic behavior in the wavefunction of the system. The smaller the E_S_, the higher the stability of the considered surface. At variance with GTOs, which allow true molecules, polymers, slab models and crystals to be defined, the delocalized nature of the PW basis also forces the adoption of a pseudo-3D system for modelling molecules, polymers and slabs. For slab surfaces, the system consists of a 3D replica of the slab of interest along a fake crystal vector perpendicular to the slab plane. The modulus of that vector should be large enough to prevent spurious interactions between the replicated slabs.

For the simulation of amorphous materials within PBC, the structural atomistic details are not available directly from experiments. One possible way out is to start from a crystalline bulk and submit the system to a simulated melt/quench process by means of molecular dynamics (MD) running at relatively high T, ensuring randomization of the nuclei positions.

While the PBC approach is very popular and also somehow computationally convenient, the available computer codes are limited to DFT as the level of theory and the tools provided to study materials and surfaces are somehow less developed with respect to computational molecular codes. It would, therefore, be particularly appealing to be able to use these codes, which allow for better treatment of difficult cases, like open-shell systems, in which CCSD(T) is desirable as the method of choice. The cluster approach, as previously described, is the “forced” path to apply sophisticated quantum mechanical methods. The usual way to prepare a cluster by extraction of a portion from the crystalline system suffers from the lost memory of the crystalline system from which it is derived. For instance, a cluster of a certain number of water molecules can be cut out from the (010) crystalline surface of ice. However, for this cluster to be representative of that surface, geometrical constraints at the frontier atoms should be enforced; otherwise, the cluster will collapse during the geometry optimization. A cleaner alternative strategy would be to adopt a large enough cluster, hoping for a natural rigidity to keep the cluster in place. This has a trade-off of increasing the calculation cost dramatically. For clusters characterized by soft intermolecular interactions, like water ice, designing a proper cluster can be very tricky. Additionally, for flexible covalent systems, like silica-based materials, the cluster approach can be rather subtle to control, as the termination of covalent bonds should also be taken into account.

A possible solution to adopt large enough clusters to ensure certain rigidity while keeping the cost of the calculation under control is the embedded cluster approach. The main idea is that the surface is structurally modelled by a large cluster model, which is described theoretically with two different levels of theory: a high level of theory for the chemically relevant part only, while a low level (and computationally cheaper) theory for the rest of the system, usually far away from the high-level zone. This defines the QM/MM embedded approach, in which the high level of theory is any quantum mechanical method (QM), while for the low level of theory, even molecular mechanics (MM) can be chosen [121]. When adopting MM as a computational method, care is taken to check for the reliability and generality of the predictions. For instance, while excellent MM force fields for water exist, their applicability to model the interaction between water and general adsorbates is highly questionable. In those cases, the low level of theory can be an SM (e.g., MNDO, AM1 or PM7) or, more recently, the tight-binding density-functional methods (like xTB-GFN2), which have been proven to be particularly good in treating intermolecular interactions between water ice and adsorbates of interest [122]. Similarly, the minimal Hartree–Fock HF-3c [123] is also a good and fast solution.

## 3. Gly Formation in the ISM

As for iCOMs, the formation of Gly in the ISM can occur through two routes: in the gas phases and on the grain surfaces. The first one advocates that the reactants are species in the gas phase, while in the second one, reactants are adsorbed and diffuse on the icy mantles. Several theoretical works have simulated Gly formation following these two major routes.

### 3.1. Gas-Phase Routes

One of the main reaction types to form Gly in the gas phase invokes the coupling of two radical species. These reactions are particularly appealing in astrochemistry because they are energetically very favorable; that is, they are barrierless, and the reaction energies are negative and very large, a crucial aspect due to the very low temperatures of the ISM. By means of these reactions, Woon [124] and Pilling [125] suggested that Gly could form by the coupling of the COOH and NH_2_CH_2_ radicals, and Sato et al. [126] suggested that Gly could form by the coupling of the H/NHCH_2_COOH, NH_2_/CH_2_COOH, NH_2_CH_2_/COOH and NH_2_CH_2_CO/OH radical pairs. Shivani et al. [127] also suggested similar processes for a plausible route to form serine, a more complex α-amino acid with R=CH_2_OH. For all the cases, the authors also investigated the formation of the reactive radicals, which were based on reactions between a simpler radical and a closed-shell species (the so-called radical-neutral reactions in the astrochemistry community). For instance, in the work of Woon [124], the formation of COOH takes place via a reaction involving OH and CO and NH_2_CH_2_ by successive reactions of H + HCN → CH_2_N, CH_2_N + H → CH_2_NH and CH_2_NH + H → CH_2_NH_2_. It is worth mentioning that the precursor radical-neutral reactions, although having very favorable reaction energies, present high energy barriers so that these previous processes can inhibit Gly formation by radical-radical couplings. The same has been seen for the reaction of CH_3_COOH with NH and NH_2_OH [125]. Additionally, the efficiency of the radical-radical coupling in the gas phase is limited since Gly can dissociate back to reactants because it is not stabilized by three-body reactions.

Ion-molecule reactions are also processes that are usually investigated in interstellar gas-phase chemistry. These reactions are based on the collision between an ion (normally positively charged) and a closed-shell species, which, to be efficient, results in the formation of two products, one of them having the charge. Adopting this kind of reactivity, Jeanvoine et al. [128], Barrientos et al. [129], and Sanz-Novo et al. [130] investigated the formation of protonated Gly (HGly^+^) by the ion-molecule reactions of (i) NH_3_OH^+^ + CH_3_COOH → HGly^+^ + H_2_O, and (ii) NH_2_OH_2_^+^ + CH_3_COOH → HGly^+^ + H_2_O, performing an exhaustive study on the possible products that result from the reactivity between the abovementioned ion/molecule pairs. In [128], additionally, chemical dynamics simulations at PM6-D were carried out, in which some initial translation energy was provided to the system. Authors found that several isomers with a chemical formula similar to that of the protonated Gly were formed, but none of them could be attributed to the actual HGly^+^.

Largo and coworkers have deeply studied several ion-molecule pathways giving rise to glycine based on the reactivity between ammonia derivatives with acetic acid, that is, NH_3_^+^ + CH_3_COOH, NH_3_^+^ + CH_2_COOH, and NH_2_^+^ + CH_3_COOH [131,132,133]. The authors fully explored and analyzed the PESs in detail via the highly correlated post-HF MP2 and CCSD(T) methods. Although the reactions were found to be exothermic, the chemical path leading to glycine has several competitive channels that hampered its efficiency.

Redondo et al. [134] also investigated at the CCSD(T) and DFT B3LYP levels, the ion-molecule chemistry between Gly and CH_5_^+^ to assess if the formation of protonated alanine (α-amino acid with R=CH_3_) was feasible. However, the reaction presented net activation energies, while the formation of other complex compounds (e.g., protonated 1-imide-2,2-propanediol) as well as CH_3_COOH + CH_3_NH_3_^+^ were processes that were more energetically favorable.

In addition to these gas-phase reactions, other works addressed the Gly formation problem by investigating other paths, the reactants of which were species usually detected in the ISM with relatively large abundances. Nhlabatsi et al. [135,136] studied the reactivity of CO + H_2_O + NH_2_CH and CO_2_ + H_2_ + NH_2_CH, with both cases giving Gly as a likely product. Among the different reaction channels elucidated, a concerted mechanism for the CO_2_ + H_2_ + NH_2_CH reaction was postulated to be the most promising one since it was suggested that (on the basis of a detailed analysis of the quantum features of the transition state) tunnelling could play an important role. Thrush et al. [137] studied the reactivity of glycolonitrile (HOCH_2_CN), which is a product of the reaction of CH_2_O and HCN, with H_2_O and NH_3_. By means of metadynamics sampling, authors constructed a free energy map of all the species that result from such a reactivity (up to 39), among them Gly. Despite the positive results as far as Gly formation is concerned, it is worth mentioning that the conditions of the reactions were not interstellar but normal (i.e., 1 bar and 298 K). Thus, the formation of such a forest of compounds in the ISM is doubtful. These reaction routes involve closed-shell species and hence high energy barriers. In this sense, Maeda and Ohno pointed out the relevance of starting from high-energy isomers as reactants to have chemical reactions with low energy barriers [138].

To sum up, several works investigating the formation of Gly under interstellar gas-phase conditions have been developed in the last years. However, none of them provide convincing evidence of the feasibility of the processes. Therefore, it is reasonable to think that synthetic routes occurring on the surfaces of the grains can be likely more efficient than the gas-phase ones. Works devoted to these reactions are described in the forthcoming section.

### 3.2. Grain Surface Routes

As mentioned in the Section 1, grains of the dense molecular clouds consist of a refractory material core surrounded by icy mantles. Because of the watery nature of the ices, a reasonable path towards interstellar Gly is that occurring in water solution, namely, the Strecker synthesis. Strecker reactions comprise the synthesis of amino acids under acidic conditions by the reactivity of aldehydes/ketones with ammonia and hydrogen cyanide. For the particular case of Gly, the reaction involves three steps:

(i) H_2_C=O + NH_3_ → NH_2_CH_2_OH → NH=CH_2_ + H_2_O;
(ii) NH=CH_2_ + HCN → NH_2_CH_2_CN;
(iii) NH_2_CH_2_CN + 2H_2_O → NH_2_CH_2_COOH + NH_3_.

This reaction is particularly attractive from an interstellar perspective because the reactants (i.e., H_2_C=O, NH_3_ and HCN) are compounds usually identified as minor species in the ice mantles, and the NH=CH_2_ and NH_2_CH_2_CN intermediates are compounds observationally detected in different interstellar environments. Because of that, different works have simulated the Strecker synthesis (partly, totally or modifications of it) for the formation of Gly in the presence of water molecules mimicking the watery ice mantles.

**Figure 1:**
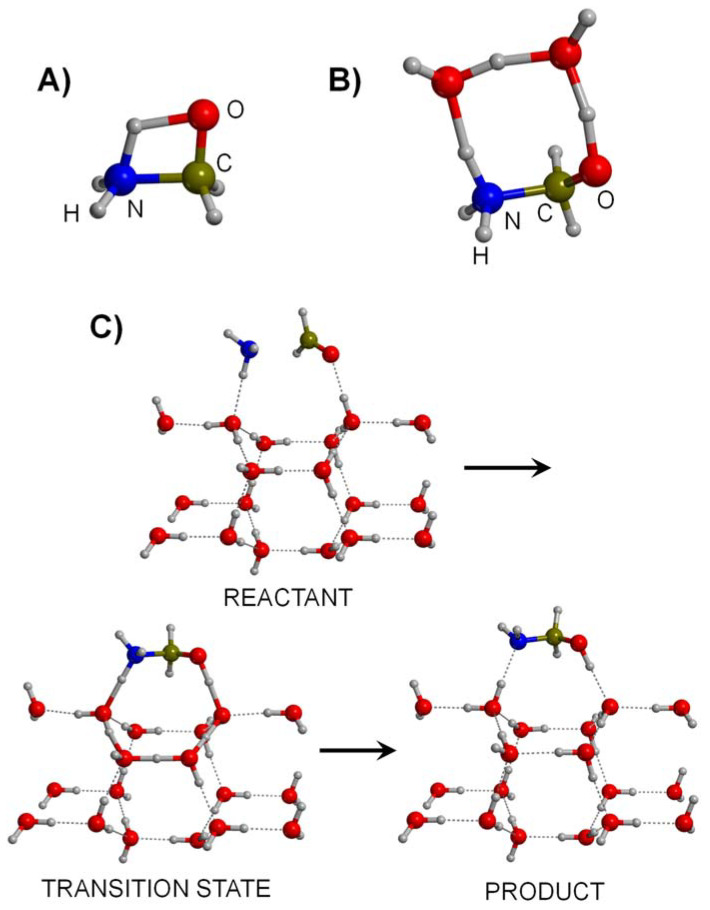
(**A**) Transition state structure of the reaction of NH_3_ + H_2_CO → NH_2_CH_2_OH (aminomethanol formation) corresponding to the first step of the Strecker synthesis under strict gas-phase conditions. (**B**) The same transition state structure as (**A**) in which two water molecules participate in the proton transfer, giving rise to a water-assisted proton transfer mechanism. (**C**) Stationary points of the aminomethanol formation on a water ice cluster model. The transition state adopts a water-assisted proton transfer mechanism involving four water molecules. Adapted from Ref. [140].

Riffet et al. [139] studied step (i) in the presence of 4 H_2_O molecules using the composite G3B3 method. The formation of NH_2_CH_2_OH was found to be thermodynamically favourable, but this was not the case for its dehydration to form NH=CH_2_. Moreover, both processes presented relatively high-energy barriers (25 and 55 kJ mol^−1^, respectively), pointing out that the processes are kinetically hampered. However, some catalytic effects were attributed to the water molecules since two of them acted as proton transfer assistants. Indeed, while in the absence of water, the transition states presented a highly strained 4th-membered ring (see Figure 1A for the formation of NH_2_CH_2_OH, with an energy barrier of 140 kJ mol^−1^), with the assistance of two water molecules, the transition states presented an 8th-membered ring (see Figure 1B), hence reducing the barrier to 25 kJ mol^−1^. This work, however, presented some limitations as far as the water ice is concerned: (i) four water molecules is a small system to realistically mimic an ice surface, (ii) the water molecules have an excess of mobility, which is not real in ice surfaces, and (iii) the capability of water to reduce the energy barriers by adopting a proton transfer mechanism can be enhanced by including more water molecules in the transition states. Overcoming such limitations, Rimola et al. [140] studied the full Strecker mechanism at the DFT B3LYP level in the presence of a cluster model made up of 18 H_2_O molecules (see the ice model of the reactant structure in Figure 1C) extracted from the crystalline Ice XI. This work showed that all the steps, when occurring on the water ice surface model, were thermodynamically favourable, since all the stepwise products were more stable than the corresponding reactants. However, the problem with this synthetic route was the kinetics. Even though all the steps were assisted by four water molecules belonging to the ice (see the transition state of Figure 1C), the energy barriers were too high (60, 73 and 163 kJ mol^−1^ for steps (i), (ii) and (iii), respectively) to be surmountable at deep space temperatures (10–20 K). However, free energy values were calculated at 100 and 200 K as these are the temperatures in hot molecular cores, regions surrounding a massive newborn star. Calculated free energy barriers did not experience significant changes (the entropic terms were almost negligible), which led the authors to suggest that steps (i) and (ii) were feasible in warmer regions (which in turn could explain the presence of NH=CH_2_ and NH_2_CH_2_CN in astrophysical environments), but step (iii), namely, the actual Gly formation, was predicted to be unlikely. Kayanuma et al. [141], by considering some of Rimola’s conclusions, studied an alternative path for step (iii) to reach Gly: the reaction between NH_2_CH_2_CN with CO_2_ (the Bücherer–Bergs reaction) to form hydantoin (2,4-imidazolidinedione, a compound identified in meteorites but not in the ISM), followed by its hydrolysis. Results computed at B3LYP and in the presence of two H_2_O molecule clusters indicated that both the formation of hydantoin and Gly were thermodynamically favourable but, once again, kinetically hindered at 10 K because of the high energy barriers of the stepwise processes (110 kJ mol^−1^ at the most). In view of these values, the authors invoked external energy sources, such as heating by radiative nuclei or shock heating in the planetesimal formation, for the occurrence of this reaction.

**Figure 2:**
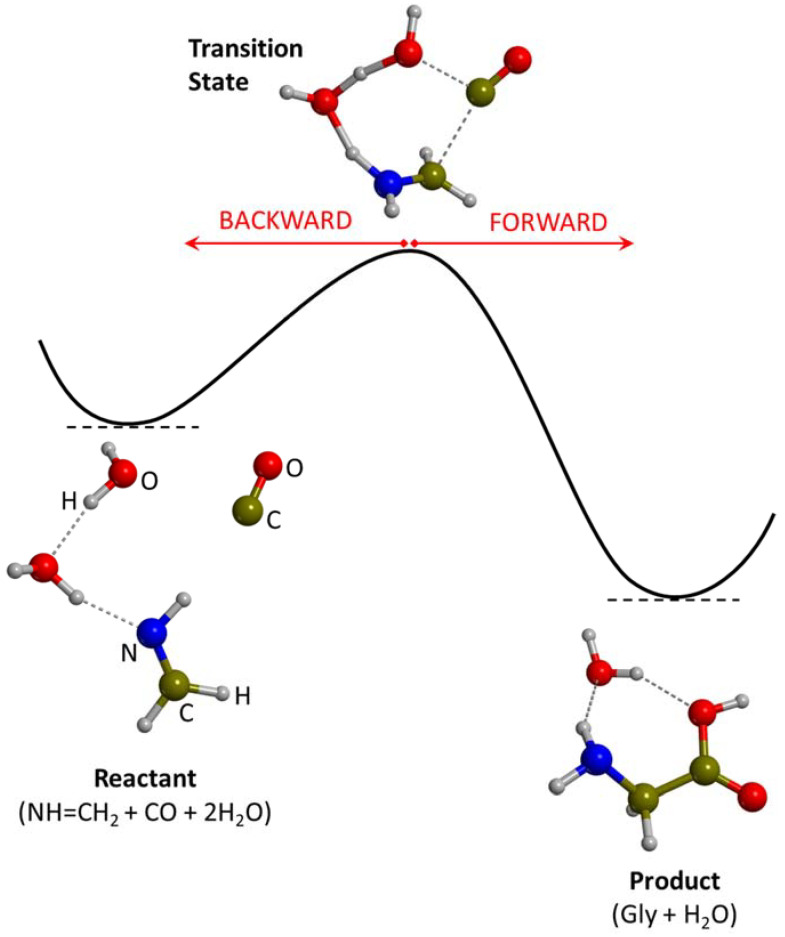
Schematic representation of the evolution towards the product (Gly + H_2_O) or the reactant (NH=CH_2_ + CO + H_2_O) in AIMD trajectories executed from the transition state. Adapted from Ref. [142].

Alternative reactions to the Strecker synthesis have also been investigated in the presence of water ice surface models. One of them is the reaction of NH=CH_2_ + CO + H_2_O → Gly, as already reported as a gas-phase route. The advantage of this reaction is that H_2_O and CO are usual components in the ice mantles, and NH=CH_2_ can be formed by energetic processing of H_2_CO+NH_3_ (see above), which are also ice mantle components. Nhlabatsi et al. [136] characterized the PES of this reaction at B3LYP in the presence of one additional water molecule acting as a proton transfer assistant. Results indicated a largely favourable reaction energy but a relatively high energy barrier (142 kJ mol^−1^), leading the authors to invoke warmer regions than cold interstellar clouds (e.g., hot molecular cores) as the location where the reaction takes place. However, in a subsequent work, Krishnan et al. [142] studied the probability of the occurrence of this reaction by means of AIMD simulations. Hundreds of trajectories from the transition state were generated over the B3LYP PES (so-called on-the-fly dynamic simulations) to check whether the system evolved forward or backward, i.e., whether Gly formed or reactants formed (see Figure 2). Results indicated that at 40 K, none of the trajectories go to Gly formation but tend toward dissociation towards the reactant side. In view that Gly formation was not possible through classical barrier crossing, the authors suggested that the reaction could be facilitated by quantum mechanical tunneling.

**Figure 3:**
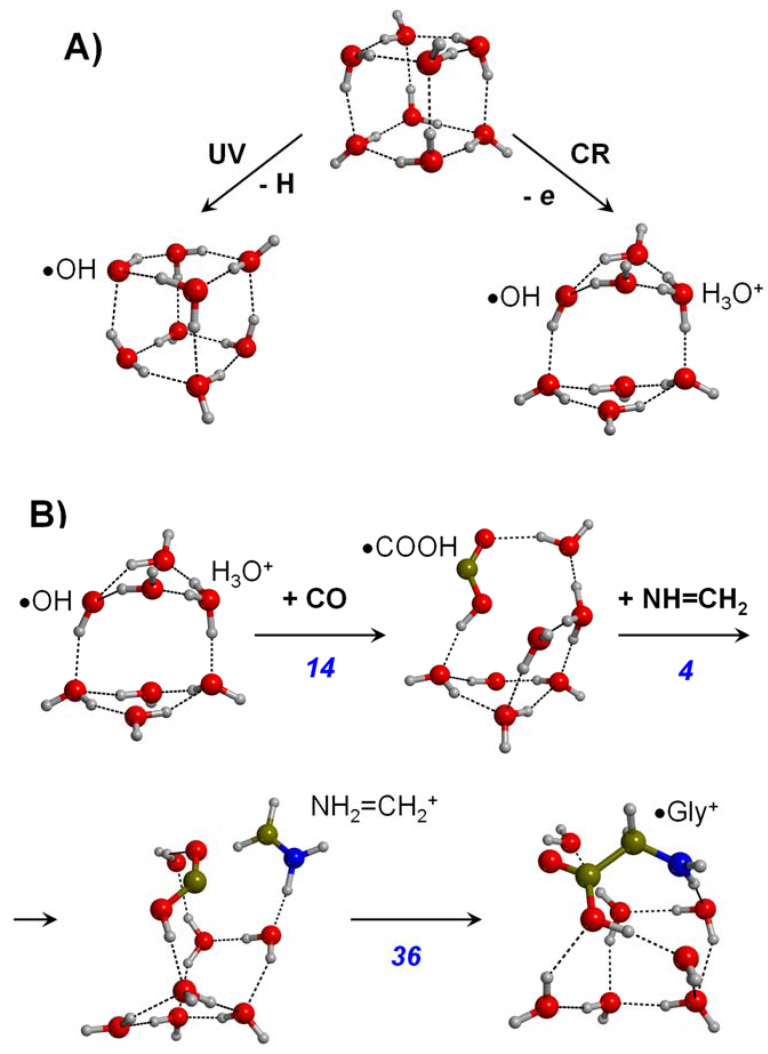
(**A**) Formation of water ice surface defects due to the incidence of ultraviolet radiation (UV) and cosmic rays (CR). (**B**) Chemical sequence to form glycine on the defective water ice cluster model. The numbers in blue are the energy barriers of each step in kJ mol^−1^. Adapted from Ref. [143].

Rimola et al. [143] studied a similar reaction but accounted for the external processing of the water ice. That is, the incidence of UV radiation to water ice usually leads to the homolytic dissociation of surface water molecules forming OH• radicals, while the incidence of cosmic rays (i.e., high energy particles), in addition to homolytic cleavages, can also lead to the ionization of the ice, in particular the formation of the OH•/H_3_O^+^ radical cation pair. These phenomena were used by the authors to study the reactivity of NH=CH_2_ + OH• + CO at the DFT BHLYP level in the presence of an 8-H_2_O cluster model containing these OH•/H_3_O^+^ defects (see Figure 3A). When adopting this water-defective cluster model, the first step was the reaction of CO with OH• to form the •COOH radical (see the first step of Figure 3B), with energy barriers as low as 13–14 kJ mol^−1^. However, the reaction of •COOH with NH=CH_2_ to form a Gly radical form (•NHCH_2_COOH) presented high energy barriers (52–54 kJ mol^−1^), hampering the reaction kinetically. Authors also simulated the possibility that the H_3_O+ cation could transfer its proton to NH=CH_2_, this way forming NH_2_=CH_2_+ (energy barrier of 4 kJ mol^−1^; see second step of Figure 3B), and that this species reacted with •COOH to form the radical cation of Gly (energy barrier of 36 kJ mol^−1^, see the third step of Figure 3B), which in turn could transform into actual Gly via electron capture (barrierless). As this sequence (shown in Figure 3B) was favorable from an energetic standpoint, the authors computed the rate constants using the classical Eyring equation at T = 10, 50, 100 and 200 K to assess its kinetic plausibility at these temperatures. Results showed that at 10–50 K, the path was kinetically very slow; in contrast, at 100–200 K, the reaction could evolve at reasonable speeds.

Another synthetic route in which relatively abundant species of the ISM are used as reactants is the reaction between HCN and H_2_O in the presence of one water. This was studied by Lee et al. [144] at the CBS-QB3 level, in which HCN transformed first into its trimer (i.e., 2HCN → NH=CHCN + HCN → NH_2_CH(CN)_2_), the hydrolysis of which could give Gly through different reaction channels. Thermodynamically, the formation of NH_2_CH(CN)_2_ and two hydrolytic paths (out of four, I and II of the original work) were found to be favorable. However, the energy barriers of all the investigated mechanistic steps, despite the great catalytic activity of the extra water molecule acting as a proton transfer assistant, were exceedingly high to occur, either in cold interstellar molecular clouds or in warmer astrophysical environments.

Kayi et al. [145] found that when CH_3_NH_2_ and CO_2_ (two commonly detected interstellar species) co-adsorbed on water ice surfaces modelled by amorphous clusters (from 0 to 20 molecules) and a crystalline cluster made up of 52 water molecules were computed at the B3LYP level of theory, the spontaneous formation of the methylcarbamic acid zwitterion, CH_3_NH_2_(+)COO(−), took place. This was due to an electron transfer from CH_3_NH_2_ to CO_2_, which induced the formation of a covalent bond between the N atom of CH_3_NH_2_ and the C atoms of CO_2_. Interestingly, CH_3_NH_2_(+)COO(−) is a zwitterionic species, the charges of which are stabilized by their interaction with the water molecules of the ice. Since CH_3_NH_2_(+)COO(−) is an isomer of Gly, authors suggested that, in view of the ease of its formation, it could be regarded as a precursor of Gly, since processing effects could induce its conversion into Gly.

Finally, reactions based on the C addition to species belonging to the ice mantles have been found as potential routes that eventually lead to glycine. The reaction of C(^3^P) + H_2_ → CH_2_ (methylene) is barrierless at B3LYP using a polarized continuum model to mimic the surface water ice effects (also tested experimentally, [146,147]). From the newly formed methylene, a different set of reactions can take place to form glycine: (i) CH_2_ + NH_2_ → NH_2_CH_2_ + CO → NH_2_CH_2_CO + OH → NH_2_CH_2_COOH, and (ii) CH_2_ + CO → CH_2_CO + OH → CH_2_COOH + NH_2_ → NH_2_CH_2_COOH [148]. The radical-radical reactions were found to be barrierless, while the radical-molecule ones exhibited low energy barriers. The reaction of C(^3^P) + NH_3_ → C-NH_3_ → CH_2_NH (methylenimine) was also found to be barrierless with respect to the asymptote at CCSD(T) [65]. CH_2_NH formation can be followed by the reactions of CH_2_NH + CO + H_2_O and CH_2_NH + CO_2_ + H_2_ (the same ones simulated by Nabhasi et al., Refs. [135,136]), leading to glycine formation. The first pathway was also tested experimentally by adding C atoms to ice clusters containing H_2_, NH_3_, and CO_2_ molecules [61]. However, it is worth mentioning that these C-addition-based reactions (both C + H_2_ and C + NH_3_) as well as the subsequent reactivity, although aimed at occurring on the grain surfaces, were simulated in their absence, namely, under gas-phase conditions.

In summary, different works related to the formation of Gly in the presence of water molecules (from very few to forming cluster systems) as a way to model interstellar water ice surfaces have been carried out. Results are overall more positive than those occurring in the gas phase. However, the most favorable paths have relatively high energy barriers, impossible to overcome at the 10–20 K of the cold, dense molecular clouds. Notwithstanding, in other, warmer regions, such as the star-forming ones and protostellar environments, the temperatures could be high enough (100–200 K) for these reactions to evolve, finally leading to the formation of Gly.

## 4. Gly Transportation and Delivery to Primitive Earth

Irrespective of the way through which Gly forms in space, the next step to evolve into a more biochemically complex form in terrestrial environments is its transportation and delivery to the primitive Earth. A feasible way was that Gly, once synthesized, was adsorbed and entrapped on asteroidal dust grains (either of cometary, meteoritic or any other origin) and then released to the early Earth’s surface. Although the chances of Gly delivery were highly likely during the epoch of intense meteoritic bombardments, it is worth mentioning that even today, great amounts of these dust grains continuously penetrate the atmosphere, enriching our planet with organic matter [149].

Theoretical simulations are useful tools to assess the validity of this “transportation and delivery” process since they can provide quantitative adsorption energies of organic compounds (here for Gly) on the grains. The critical point is what sort of solid materials one has to consider in this Gly adsorption. The rocky components of comets, meteorites and other asteroidal bodies are highly complex and heterogeneous (for instance, in meteorites, more than 275 class of minerals have been identified [150]. However, the most usual and recurrent families are: (i) silica, silicates, and aluminosilicates, (ii) metal oxides and (iii) metal sulphides. Because of that, we report here the interaction of Gly with different materials belonging to these families. As a final comment, it is worth mentioning that these materials are usually in an amorphous structural state. However, due to the difficulty to model theoretically amorphous materials (see above), in most of the reported results, the solid-state substrates are in a crystalline form, in which the interaction occurs on extended surfaces (usually the most stable one) of their crystal morphology. Although this is not the actual situation, results provide useful insights into the Gly/surface interactions, as they are essentially driven by the presence of local surface defects (e.g., unsaturated sites, vacancies, dangling bonds), which are available in both crystalline and amorphous states.

### 4.1. Gly Interaction with Silica, Silicates and Aluminosilicates

These materials are abundant not only in the nuclei of comets, meteorites and interstellar grains but also in the Earth’s crust. Pure silica (SiO_2_) consists of [SiO_4_] tetrahedral units, whose different possible arrangements define a wide variety of polymorphs and structures. Silica surfaces are featured by siloxane (Si-O-Si) and silanol (SiOH) groups, the relative abundance of which dictates their adsorbent properties. Silicates are SiO_2_-based materials in which the negative charges of the [SiO_4_]^4−^ units are compensated by divalent cations (usually Mg^2+^ and Fe^2+^). Olivines are one of the most important silicate families, with the general formula Mg_2x_Fe_(2−2x)_SiO_4_ (x = 0–1), whose Mg-pure member is forsterite (Mg_2_SiO_4_). Aluminosilicates are silicates in which Si atoms have been morphologically substituted by Al atoms. They usually contain additional alkali and alkaline earth cations and are a major component of clay minerals, such as montmorillonite.

**Figure 4:**
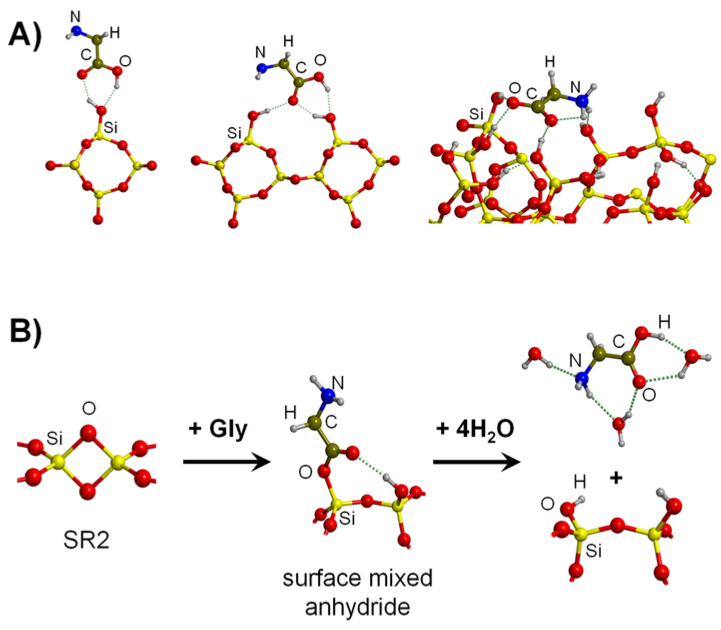
(**A**) Different complexes formed from the interaction of Gly with silica surfaces (adapted from Ref. [119]). (**B**) Reaction of Gly with silica S2R surface defect to form a surface mixed anhydride, and the subsequent release of Gly by the action of water (adapted from Ref. [151]).

Gly adsorption on silica surfaces has extensively been reviewed in a work by some of us [119]. Due to the different silica structures alongside the type of SiOH groups (i.e., isolated SiOH, geminal HOSiOH or interacting SiOH···SiOH), there is not a unique stable complex for Gly/silica. Gly adsorption in pure gas-phase conditions can be due to the interaction with one or several SiOH groups (some of them are shown in Figure 4A). Irrespective of the kind of Gly/silica complexes, it is worth mentioning that, for all cases, the adsorption energies are actually favorable, spanning the −40–−140 kJ mol^−1^ range. Additionally, calculations also indicated that Gly chemisorption could also take place on silica surfaces if they contain strained ring defects. Indeed, Rimola et al. [151,152] showed that surface (SiO)_2_ ring defects (see SR2 structure of Figure 4B as a cluster model) are highly reactive when in contact with Gly (and with COOH-containing molecules [153]), forming the so-called surface mixed anhydride Si_surf_–O–C(=O)–CH_2_NH_2_ (see the first step of Figure 4B). Moreover, the authors modelled the hydrolysis of the surface mixed anhydride by its reaction with 4 H_2_O molecules, leading to the release of Gly (see the second step of Figure 4B), with the results indicating that the process is energetically feasible under prebiotic conditions (free energy barrier and reaction energy at 298 K of 88 and −60 kJ mol^−1^, respectively, at B3LYP level). That work clearly pointed out the feasibility of defective silica surfaces in first capturing and later delivering cosmic Gly to the early Earth.

**Figure 5:**
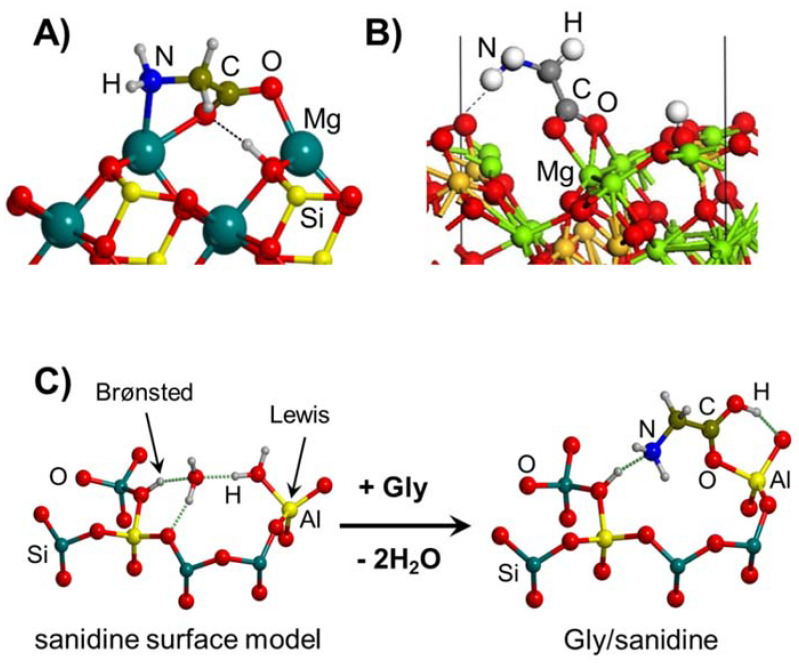
(**A**,**B**) Interaction of Gly with a silicate surface adopting different adsorption modes (adapted from Refs. [154,155]). (**C**) Sanidine surface model containing an acidic Lewis and Bronsted site, and the interaction of Gly with these surface sites (adapted from Refs. [158,159]).

In relation to Gly adsorption on periodic slab models of silicates, works on Mg_2_SiO_4_ surfaces by Rimola et al. [154] and Escamilla-Roa et al. [155] indicated that, although the Gly adsorption mode depended on the surface plane (i.e., on the (101) surface Gly adsorbed through an (N,O,O) mode and on the (100) one through an (O,O) mode; see Figure 5A,B), the adsorption presented common features: (i) the interaction was essentially between surface unsaturated Mg atoms and Gly electron donor atoms, and (ii) Gly became spontaneously deprotonated upon adsorption, forming a Gly(−)/surface(+) ion pair. Computed adsorption energies were found to be largely favourable: −320 kJ mol^−1^ on the (101) surface (at the B3LYP-D2 level using GTOs) and −402 kJ mol^−1^ on the (100) surface (at the PBE level using PWs). Escamilla-Roa et al. also studied Gly adsorption in the presence of thin layers of H_2_O [156] and H_2_O/NH_3_ mixtures [157]. In both cases, simultaneous adsorption of Gly with the solvent layers was more favourable than Gly on top of the layers.

Finally, Gly adsorption on aluminosilicates has also been reported. In a couple of works by Rimola et al. [158,159], Gly was adsorbed on a cluster model of an acidic sanidine feldspar containing both a coordinatively unsaturated Al atom and a proton in close proximity (see the surface model of Figure 5C), corresponding to acidic Lewis and Brønsted sites, respectively. Gly/sanidine interaction, simulated at the B3LYP-D2 level, occurred through both sites: the COOH group with the Al atoms and the NH_2_ group with the proton (see the complex of Figure 5C). Adsorption energies were not reported, but the favorable reaction energy for the displacement of two water molecules initially adsorbed on the acidic Lewis and Brønsted sites (about −15 kJ mol^−1^, see the process of Figure 5C) pointed out a very favorable Gly/sanidine interaction. Escamilla-Roa et al. [160] simulated the adsorption of Gly in the interlayer regions of a K+-montmorillonite clay. Under strict dry conditions, Gly was adsorbed in its zwitterionic form, i.e., NH_3_(+)CH_2_COO(−), with an adsorption energy of about −84 kJ mol^−1^, and in the presence of 20 water molecules, adsorption was even more favorable (about −197 kJ mol^−1^) keeping its zwitterionic state.

### 4.2. Gly Interaction with Metal Oxides and Sulphides

Among metal oxides identified in presolar grains (i.e., solid matter that originated before the Sun was formed, including interstellar stardust, cometary and meteoritic dust, and interplanetary dust particles), the most usually identified ones are Al_2_O_3_, MgAl_2_O_4_, CaAl_12_O_19_ and TiO_2_ [161,162,163,164]. Interestingly, TiO_2_ has been invoked as, in addition to silicate, a likely candidate to first form under the astrophysical conditions at which interstellar dust nucleated and condensated [165,166]. In relation to metal sulphides, FeS has been identified in protoplanetary disk grains [167] and is common in the mineralogy of meteorites [168]. Moreover, its presence indicates a low degree of water alteration since magnetite (Fe_3_O_4_) is the associated Fe-containing mineral for water-altered meteorites [42]. In comets, the usual way to identify iron sulphides is due to the presence of GEMS, i.e., glass with embedded metal and sulphides [169,170]. Investigations on the mineralogical nature of the 81P/Wild 2 and 67P comets indicate that the metal sulphide content is in the form of FeS and Fe-Ni sulphides [171,172,173,174,175].

**Figure 6:**
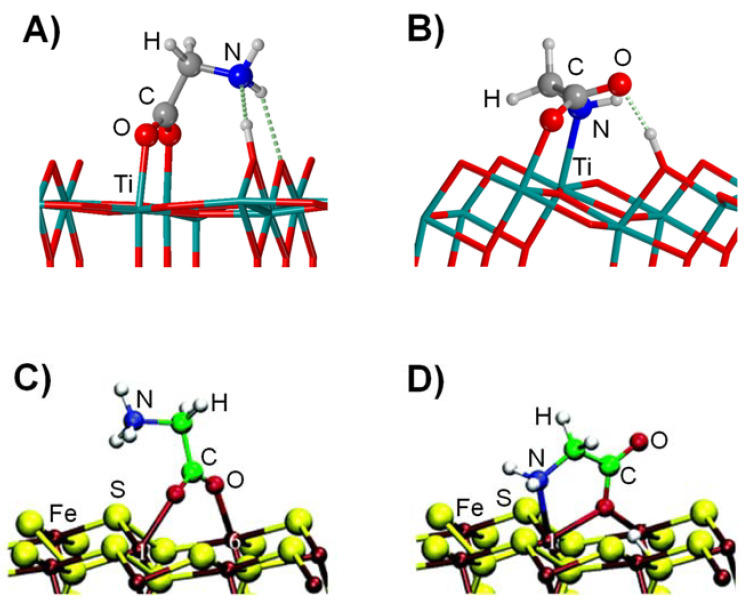
Interaction of Gly with the rutile (110) and the anatase (101) TiO_2_ surfaces (**A**,**B**), respectively, adapted from Refs. [180,179]), and with a vacancy-defective (100) FeS_2_ surface (**C**,**D**), adapted from Ref. [181]).

Several theoretical works have reported the interaction of Gly with TiO_2_ surfaces [176,177,178,179,180], in particular with the rutile (110) and the anatase (101) ones, the most stable crystal faces of these two naturally occurring TiO_2_ polymorphs. On both periodic slab surfaces, the most stable form of Gly is in its deprotonated form (see Figure 6A,B) over the zwitterionic and canonical forms. Differences in the Gly/TiO_2_ complexes due to the surfaces are that (i) on the rutile surfaces (110), Gly adopts an (O,O) binding mode, while on the anatase surfaces (101), Gly adopts an (N,O) binding mode, and (ii) the adsorption energies are significantly larger on the rutile surfaces than on the anatase surfaces (e.g., at PBE-D2*/PWs theory level, −238 and −108 kJ mol^−1^, respectively) [179,180]. The difference in the adsorption modes is due to the Ti-Ti distances: those of the rutile (110) match very well with the O-O Gly distances, while those of the anatase (101) are longer, making the simultaneous interaction with the Gly N and O atoms more favourable. The difference in the magnitude of the adsorption energies is due to the stabilities of the surfaces (determined by the surface energies, E_S_): the rutile (110) surface is less stable than the anatase (101) one (E_S_ = 0.80 and 0.37 J m^−2^, respectively), and accordingly, the former is more reactive than the latter, hence presenting larger adsorption energy values. Despite these differences, these data clearly show that TiO_2_ surfaces are suitable substrates to capture cosmic Gly.

Finally, works addressing Gly interaction on pristine FeS surfaces have not been reported yet. In contrast, Gly interaction on a sulphur vacancy-defective (100) FeS_2_ periodic surface model (which could locally render an FeS surface) is available [181]. On this surface, two complexes were identified as stable (computed at PBE/PWs), exhibiting either (O,O) or (N,O) adsorption modes (see Figure 6C,D). An additional interesting aspect of this work was that the authors also investigated the Gly desorption using the metadynamics technique due to the effect of hot-pressurized water (resembling the conditions of deep-sea hydrothermal vents). Results indicated that the full desorption of Gly was a more stable situation than the complexes (about 200 kJ mol^−1^ more stable) and that desorption presented an activation energy of about 90 kJ mol^−1^, indicating Gly retention times on the surface of milliseconds. These results pointed out that Gly, once it reached the primitive Earth surface, could have been released from the surface due to the action of water, in a similar way as the case of the SiO_2_ strained ring defects (see above).

## 5. Gly Polymerization in the Primitive Earth

**Figure 7:**
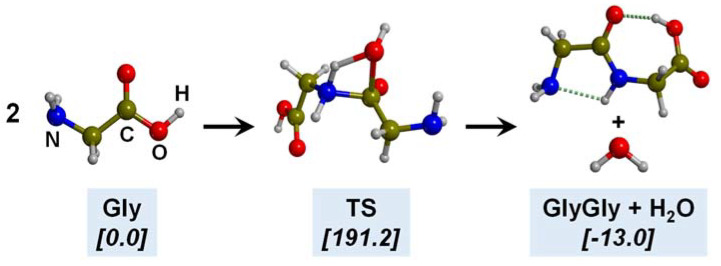
Reaction mechanism of the peptide bond formation between two Gly molecules. TS is the transition state connecting Gly with Gly Gly. Values in brackets refer to the energetics of the process in kJ mol^−1^.

The polymerization of Gly leading to the formation of peptides takes place by the condensation reaction between two Gly molecules, i.e., 2NH_2_CH_2_COOH → NH_2_CH_2_CH(=O)NHCH_2_COOH + H_2_O (see Figure 7 for its intrinsic reaction mechanism in the gas phase). The chemical link between the two Gly (and, by extension, between two amino acids) is the peptide bond CH(=O)-NH. This reaction is associated with the release of one water molecule. This water release, however, renders the reaction to be thermodynamically disfavoured in an aqueous solution due to Le Chatelier’s principle. Additionally, the intrinsic gas-phase reaction presents a high free energy barrier (≈190 kJ mol^−1^ in normal conditions, computed at B3LYP-D3) due to the highly strained 4th-membered ring present in the transition state structure (see Figure 7), and it is nearly isoergonic. Several postulates overcoming such thermodynamic and kinetic problems have been investigated, including the role of mineral surfaces, the presence of iron sulphides in extreme oceanic conditions, and the interaction of metal cations in aqueous environments as promoters of the reaction. These three plausible scenarios are briefly explained in the following subsections jointly with investigations carried out with quantum chemical simulations supporting them.

### 5.1. In the Presence of Mineral Surfaces

The formation of peptides in the presence of mineral surfaces was first suggested in the middle of the last century by the British biophysicist J. D. Bernal. He proposed the possibility that minerals can concentrate amino acids and activate them to polymerize and protect the formed peptides from external actions such as hydrolysis [182]. This seminal postulation has been subsequently strengthened and improved by a great number of experimental investigations [183,184,185,186,187,188,189,190,191,192,193,194,195,196,197,198,199], not only for peptide formation but also for the formation of other relevant biochemical systems such as polynucleotides [200,201]. The key point of this “polymerization on the rocks” [202,203] is that minerals can thermodynamically and kinetically favour the condensation of the biomolecular building blocks because, in essence, (i) mineral surfaces can act as dehydrating agents retaining the released water on their surfaces (thus overcoming the thermodynamic problem) and (ii) the interaction of the reactants with the mineral surfaces can activate them and reduce the energy barriers (thus overcoming the kinetic problem).

**Figure 8:**
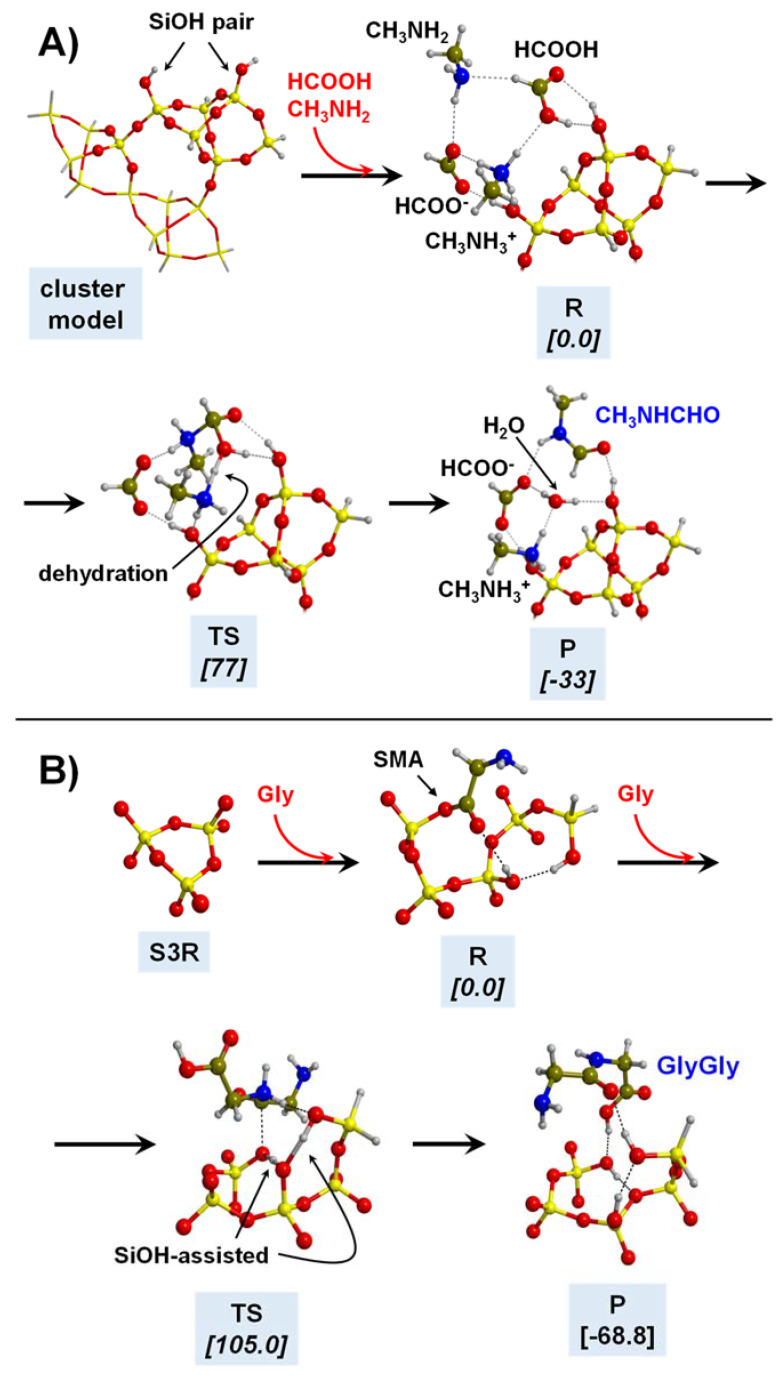
(**A**) Mechanism of the condensation reaction between HCCOH and CH3NH_2_ catalysed by silica surface represented by an amorphous cluster model (adapted from Ref. [189]). (**B**) Mechanism of the condensation reaction between two Gly molecules in the presence of the S3R surface defect (see text), in which a surface mixed anhydride (SMA) complex is formed as an intermediate, and the condensation is catalysed by a silanol (SiOH)-assisted proton transfer mechanism (adapted from Ref. [152]). R means the reactants, TS the transition state, and P the product. Values in brackets refer to the energetics of the process in kJ mol^−1^.

Silica-based minerals are among the most abundant inorganic materials in Earth’s crust. Here we provide three theoretical works to illustrate the potentiality of these minerals to polymerize Gly. In a combined experimental and theoretical study, Rimola et al. [189] found that pure silica surfaces can help the condensation reaction between CH_3_NH_2_ and HCOOH (in this work, used as a reaction model between two Gly molecules). IR measurements elucidated that the presence of specific weakly interacting SiOH pairs was key for the reaction. This phenomenon was rationalized by theoretical calculations and using a cluster model for silica as follows: the weakly interacting SiOH groups (which stay ≈ 5 Å apart; see Figure 8A) were sites that allowed the adsorption of the reactants in both their canonical and ionic states (i.e., CH_3_NH_2_/HCOOH and CH_3_NH_3_^+^/HCOO^−^, respectively; see Figure 8A). This coexistence was fundamental for the occurrence of the reaction because the canonical pair was the reacting one while the ionic pair acted as the actual catalyst (see Figure 8A), giving a free energy barrier (computed at B3LYP-D3 at T = 323 K, that of the experiments) of 77 kJ mol^−1^. Moreover, the reaction free energy was −33 kJ mol^−1^, in which the released water was retained by H-bonds with the surface (see Figure 8A). In another work, Rimola et al. [152] showed that the surface SiOH groups can have an active role as catalysts in the Gly condensation reaction. Indeed, in this work, silica rings of moderate strain, namely, (SiO)_3_, so-called S3R, present in a silica cluster model can react with Gly to form a surface mixed anhydride (SMA, see above and Figure 8B), which in turn can react with an incoming Gly molecule. However, for the occurrence of the reaction, surface SiOH groups have to assist the initial proton transfer from the nucleophile NH_2_ group (see Figure 8B), thus reducing the B3LYP-D3 free energy barrier (T = 298 K) to 105 kJ mol^−1^. Moreover, since the reaction does not release water but forms a new SiOH group (see Figure 8B), the reaction free energy was largely favourable at −68.8 kJ mol^−1^.

**Figure 9:**
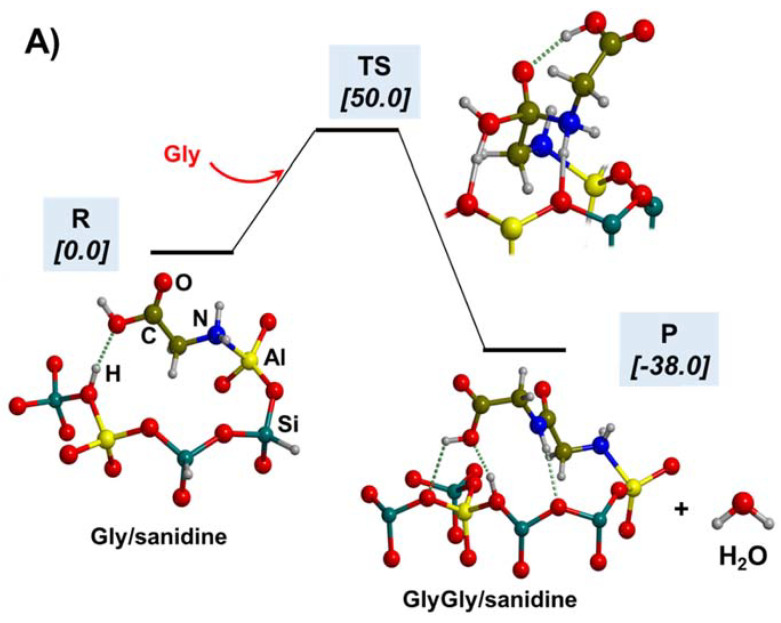

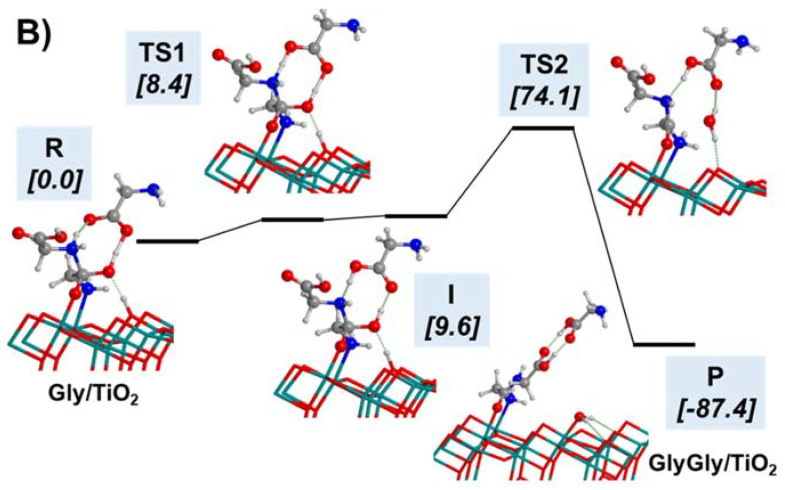
Free energy profiles at 298 K (in kJ mol^−1^) for the condensation reactions between two Gly molecules on a sanidine surface cluster model ((**A**), adapted from Refs. [158,159]) and on the anatase (101) TiO_2_ surface model ((**B**), adapted from Ref. [207]), with this latter case occurring in the presence of a third Gly molecule that catalyses the processes. R means the reactants, TS the transition states, P the products, and I the intermediate.

In addition to silica, aluminosilicate surfaces have also been proven to facilitate peptide bond formation. This is the case of the works by Rimola et al. [158,159] using a sanidine feldspar surface model that exhibits Lewis and Brønsted acidic sites (shown above, see Figure 5C). These works indicated that the two acidic sites were important for the reaction because (i) the Lewis site activates the CCOOH atom of the adsorbed Gly to be more prone to the nucleophilic attack of the second Gly, and (ii) the Brønsted site catalyses the dehydration process by transferring its H^+^ to the OH group (see Figure 9A). Moreover, the localized transition state revealed a synchronism that further reduced the energy barriers: the NH_2_ group of the second Gly transferred one of its H^+^ to the surface during the nucleophilic attack, while the Brønsted site transferred its H^+^ to carry out the dehydration (see TS structure of Figure 9A). The overall process presented highly favorable energetics, with a B3LYP-D2 free energy barrier and a reaction free energy (T = 298 K) of 50 and −38 kJ mol^−1^, respectively. In addition, it was also shown that the formed peptide, which remained attached to the surface, was resistant to hydrolysis, since the process was calculated to be endergonic (+54 kJ mol^−1^). The authors also demonstrated that the peptide bond formation can be repeated several times to form an actual polypeptide.

Earth’s crust also contains certain minerals, which despite not being actually abundant, possess very attractive catalytic properties. This is the case of TiO_2_, whose exclusive physicochemical and catalytic features have been extensively investigated in industrial and technological fields. The role of TiO_2_ as an important mineral in prebiotic chemistry has also been addressed elsewhere [204,205,206]. In the particular case of Gly polymerization, Martra et al. [188] identified by means of IR and mass spectrometry techniques the formation of polyglycine peptides on anatase TiO_2_ nanoparticles when monomers were successively introduced in the reaction chamber. Pantaleone et al. [207] interpreted these experiments by means of PBE0-D2/PWs calculations simulating the condensation between two Gly molecules on the periodic TiO_2_ (101) anatase surface under strict gas-phase conditions. There, it was found that Gly interaction with the TiO_2_ surface exerted some catalytic effect but not enough to overcome the energy barrier at normal conditions (≈150 kJ mol^−1^). In fact, the actual catalytic effect was exerted by a third Gly molecule if it directly participated in the initial proton transfer (see Figure 9B), reducing the free energy barrier of this step to 10 kJ mol^−1^. The bottleneck of the reaction was the dehydration step (free energy barrier of 70 kJ mol^−1^, see Figure 9B), in which the released water molecule was adsorbed on the TiO_2_ surface, rendering a reaction free energy as favorable as −90 kJ mol^−1^.

### 5.2. In the Presence of Iron Sulphides under Oceanic Extreme Conditions

Iron sulphides have also been invoked as relevant materials in the evolution of the primordial chemistry, in this case in the framework of the chemoautotrophy theory developed by Wächtershäuer [208] and Russell [209]. This theory proposes that the catalytic properties of iron sulphides driven by their redox chemistry could have promoted C fixation reactions from, e.g., simple CO or CO_2_ that allowed the formation of organic compounds of biological relevance. These bioorganic compounds, on the iron sulphide surfaces, could have served as catalytic centres where primordial metabolic processes might operate, giving rise to the so-called autocatalytic surface metabolism. Hydrothermal vents present on the seabed, including their extreme conditions (i.e., high temperatures and pressures), have been shown to be plausible environments in which this surface metabolism can work [210]. A fundamental aspect of this theory is that the redox energy released in the oxidation of FeS to FeS_2_ at the expenses of the reduction of H_2_S into H_2_ (ΔG = −38.6 kJ mol^−1^) could have promoted the C fixation reactions. This has been validated by experimental evidence, e.g., formation of carboxylic acids from the reaction between CH_3_SH and CO on (Fe,Ni)S surfaces [211,212], the formation of amino acids by the reaction of CO with cyano and methylthio ligands bound to (Fe,Ni)S surfaces [213], and the formation of peptides by activation of amino acids with CO in the presence of H_2_S/CH_3_SH on (Fe,Ni)S surfaces [214,215].

**Figure 10:**
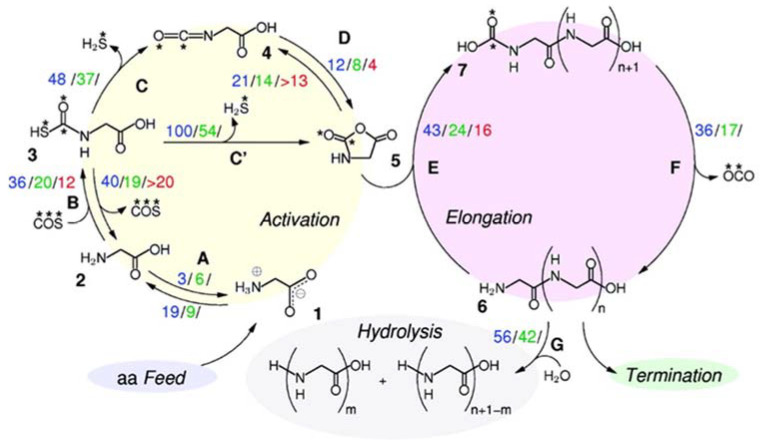
Results obtained through metadynamics for the peptide bond formation between two Gly molecules activated by COS and occurring under extreme conditions (hot pressurized water) on the FeS_2_ surface. Units are in k_B_T. Stars labels the atoms arising from COS. Reprinted with permission from J. Am. Chem. Soc. 2008, 130, 9, 2768–2770. Copyright 2018 American Chemical Society.

D. Marx and co-workers have long been devoted to investigating the formation of peptides on FeS_2_ surfaces under the extreme conditions of hydrothermal vents [181,216,217,218,219,220]. To carry out these investigations, metadynamics simulations were adopted to obtain free energy landscapes using the PBE/PWs methodology of the reactions at high T and P (namely, 500 K and 20 MPa). All these referenced works pointed out a favourable condensation of two Gly molecules, in which, crucially, previous to the reaction, one Gly was activated by COS forming the activated intermediate of N-carboxyanhydride (see the scheme of Figure 10). Results indicated that both the extreme conditions and the FeS_2_ surface were key for the progress of the reaction (compared to ambient bulk water). That is, extreme conditions allowed the stabilization of the canonical form of the Gly molecules (essential since Gly zwitterion forms are highly inactive toward polymerization) because hot pressurized water has a dielectric constant of ≈6. Simultaneously, pyrite acted as a catalyst reducing the free energy barrier, particularly the entropic contribution, because of the immobilization of the reactants on the surface. Additionally, plausible hydrolysis of the formed peptide was also investigated, with the results indicating substantially higher free energy barriers than those of the peptide formation.

In a set of subsequent works, D. Marx and coworkers studied at PBE/PWs the formation of peptides under nanoconfined water conditions [221,222,223], i.e., liquid water confined in nanometric spaces, which were defined by two terminated FeS sheets. The idea was to elucidate possible nanoconfinement effects since nanoconfined liquids can affect chemical reactions due to their different structural and dynamic properties with respect to the bulk systems. In these works, reactions leading to the formation of peptides under extreme nanoconfined conditions were simulated using metadynamics simulations. Results indicated important differences with respect to FeS-free hot pressurized bulk water, among them (i) the geometry constraints imposed by the nano-space, in this case properly orienting the reactants, and (ii) the capability of the interfacial water to stabilize exclusive intermediate charged species, which facilitated the occurrence of the overall process.

### 5.3. In the Presence of Metal Cations in Aqueous Solution

The experimental evidence that systems containing amino acids evolve into peptides in an aqueous solution in the presence of relatively concentrated NaCl and a small amount of CuCl_2_ at T ≈ 80 °C [224] formed the basis of the “salt-induced peptide formation” (SIPF) theory. Under these conditions, it was postulated that (i) the sodium ions are not fully hydrated so that they can act as strong dehydrating agents, and (ii) the interaction of the reactive amino acids with Cu^2+^ brings the two partners in close proximity and activates them, hence reducing the activation energies [225,226]. The SIPF reaction has also been combined in the presence of clay minerals, improving the yields of the peptide formation [227,228]. Despite these findings, the SIPF reaction has never been fully simulated with quantum chemical calculations.

**Figure 11:**
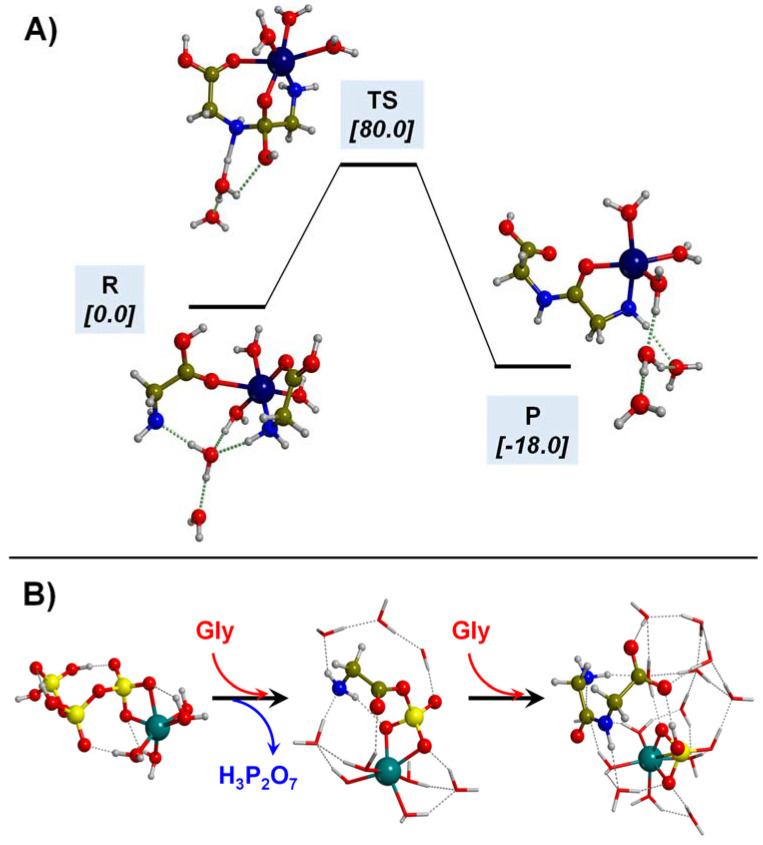
(**A**) Free energy profile at 298 K (in kJ mol^−1^) in the presence of Cu^2+^ and in water solution (adapted from Ref. [229]). (**B**) Schematic representation of the peptide bond formation between two Gly molecules intermediated by the formation of phosphoglycine (species at the centre of the sequence) in the presence of Mg^2+^ cation and in water solution (adapted from Ref. [230]).

An interesting point of the SIPF reaction is the activation of the condensing amino acids when they interact with Cu^2+^. This inspired a theoretical work of Rimola et al. [229] to get deeper insights into this aspect of Gly condensation (see Figure 11A). It was found that both the interaction with Cu^2+^ and the presence of water solvent molecules were important to reduce the free energy barriers (about 80 kJ mol^−1^ in normal conditions at BHLYP). Indeed, the Gly/Cu^2+^ interactions enhanced the electrophilic character of the CCOOH atom of one Gly to be more prone to the nucleophilic attack of the NH_2_ of the other Gly, while solvent water molecules acted as proton transfer assistants in the dehydration step (see Figure 11A), thus catalysing the proton transfer from the NH_2_ to the OH groups.

Finally, the role of Mg^2+^ cations in the peptide bond formation was investigated by Martínez-Bachs et al. [230], adopting a Gly phosphorylation mechanism. Here, the reaction consisted of two steps: (i) phosphorylation of one Gly to form phosphoglycine (an activated intermediate), and (ii) the condensation between this intermediate and a second Gly molecule (see Figure 11B). The idea was to assess if a similar mechanism of peptide synthesis by amino acid condensation occurring in living organisms (in which phosphorylation is carried out by ATP) could have operated in primordial watery environments in which triphosphates (the phosphorylation source) and Mg^2+^ cations were diluted. In this work, Mg^2+^ was not directly interacting with Gly but with triphosphate, which remarkably was found to be important for the formation of phosphoglycine. However, the actual condensation between phosphoglycine and the second Gly was found to be kinetically hindered (free energy barrier of about 170 kJ mol^−1^ at B3LYP-D3) due to the enhanced stability of the pre-reactant complex caused by solvent effects. Despite these negative results, the authors opened up the possibility that similar reactions could occur in the interlayer clay regions, since the presence of water is limited.

## 6. Conclusions

The present work is a review of the available studies based on quantum chemical computations on the chemical origin and evolution of Gly, which are consistently joined together following a ladder of organization events that trail a likely Gly’s life, that is, from its synthesis in the ISM, its carriage in cometary and meteoritic material and exogenous delivery on the early Earth, up to its transformation into peptides in a prebiotic era.

Relative to the Gly synthesis in space, works based on processes occurring in the gas phase and on the icy grain mantles have been reviewed. The works provided molecular insights into the proposed synthetic pathways and the related energetics, which in some cases were complemented with kinetic calculations. Although most of the routes were found to be promising, they required additional inputs for proper progress, such as the need for some temperature (100–200 K) to activate the reactions, the presence of third bodies that dissipate the large nascent energies, the occurrence of quantum tunnelling, or the presence of water ice defects caused by UV/cosmic-ray incidence.

According to the abovementioned sequence, the next step is the transportation of Gly to the primitive Earth by fragments of asteroids (found in meteorites) or comets (found in interplanetary dust particles), which could well have occurred during the very first phases of the solar system formation [231]. On that, theoretical works mostly address the interaction of Gly with different minerals present in comets and meteorites, particularly silica, silicates, aluminosilicates, metal oxides, and metal sulphides. The interaction of Gly with these inorganic materials is actually very strong, demonstrating their capability to capture and retain Gly up to an eventual release on Earth. Moreover, in some works, such a release process was demonstrated to be carried out by water, e.g., of the primitive Earth’s oceans.

Finally, peptide formation from the condensation of two Gly molecules has been reviewed. Here, the reactions have been focused on those catalysed mineral surfaces and on those occurring in the presence of metal cations in aqueous solution, which belong to the “polymerization on the rocks” and “salt-induced peptide formation” frameworks, respectively. On the one hand, it is shown that the peptide bond formation is favourably catalysed by different mineral surfaces relatively abundant on the Earth’s crust, such as silica, aluminosilicates and TiO_2_, the origin of the catalytic effect being exclusive to each material. Condensation reactions on iron sulphide surfaces under extreme conditions (i.e., hot pressurized water) have also been shown to be favourable thanks to the interplay of the surfaces and the extreme conditions. On the other hand, reactions in the presence of Cu^2+^ cations under normal watery conditions could have also contributed to the formation of the first peptides. Additionally, condensation reactions intermediated by the formation of phosphoglycine (due to a reaction between one Gly and triphosphate), in which the Mg^2+^ cation acts as a holder of the reactive species, have been shown as a promising synthetic route, rather than in aqueous solution, in the dry interlayer regions of clay minerals.

In view of all these results, collected here as a comprehensive review, a lot of work has been done. However, there is still a large space to further investigate the primordial trail of Gly’s life.

Most of the works dealing with the formation of Gly in the ISM are based on chemically intuitive synthetic pathways (namely, the authors think of a plausible formation route to simulate), which probably implies that other, possibly efficient different paths are neglected. Thus, the automation of the tasks to identify chemical routes towards Gly formation (and other biomolecular building blocks) will be truly useful, such as the transition state search using chemical dynamics simulations (TSSCDS) [232,233] and the artificial force induced reaction (AFIR) [234,235,236] methods, which obtain transition state structures and related reaction paths in an automatic way.

Despite the clear evidence that Gly is present in comets and routinely identified in meteorites (as a volatile compound), the simulation of its synthesis on solid-state phases of these bodies is missing. Here, in addition to the atomistic insights of the different chemical routes (mechanistic steps and related energetics), it will be of paramount interest to determine if the mineral surfaces play any essential catalytic role and to assess the relevance of hydrothermal alteration, irrespective if it is direct (i.e., the reactions become favourable due to the presence of “hot” water) or indirect (i.e., the reactions are only possible due to the presence of minerals that can only be formed upon thermal water alteration).

Available computational studies show that minerals present in asteroidal bodies can indeed carry Gly, but very few studies have been done in relation to its delivery. Determining the energy features associated with the desorption of Gly from the carrier materials immersed in water solution is of great interest to understand the feasibility of the process and if water is crucial in driving this delicate exogenous delivery step. AIMD simulations adopting the potential of mean constraint force (PMF) method, which allows how the free energy changes as a function of Gly/surface distance to be known, hence simulating the Gly desorption process, will be of high value to this end. Furthermore, works dealing with the glycine/surface interactions from a vibrational spectroscopic standpoint will also contribute to the topic, since the vibrational features and their perturbations due to the adsorption can provide hints, particularly of the structures of the complexes, on the binding of glycine on the surfaces. To this end, however, the inclusion of anharmonicity in the calculations can be crucial to accurately predict the spectroscopic properties of isolated and chemisorbed molecules, as found for glycine in interaction with the silicon surface [237,238].

Finally, a suite of theoretical works dealing with Gly condensation under prebiotic conditions has been developed. Nevertheless, relative to this subject, there is still an important gap to be filled, i.e., Gly polymerization in clays. This is actually surprising since the original Bernal’s hypothesis mainly advocated clays as the mineral activators of peptide formation, which is still valid and reinforced in view of that fact that layered double hydroxides (an important class of layered clays) could indeed have played an important role in these reactions [239].

All in all, therefore, it seems clear that there is still a fertile ground to investigate the primordial chemical life of Gly to have deeper insights into this topic that, due to its interdisciplinary nature and fundamental character, attracts the attention of scientists of different fields and broad audiences.

## Data Availability

Data is contained within the article.
